# Use of Real-Time Polymerase Chain Reaction to Differentiate between Pathogenic *Entamoeba histolytica* and the Nonpathogenic *Entamoeba dispar* in Ecuador

**DOI:** 10.4269/ajtmh.17-1022

**Published:** 2018-11-05

**Authors:** Ángel Guevara, Yosselin Vicuña, Denisse Costales, Sandra Vivero, Mariella Anselmi, Zeno Bisoffi, Fabio Formenti

**Affiliations:** 1 Carrera de Medicina, Instituto de Biomedicina, Universidad Central, Quito, Ecuador;; 2 Laboratorio Clinico, Hospital General Docente Calderón MSP, Quito, Ecuador;; 3 Centro de Epidemiología Comunitaria y Medicina Tropical (CECOMET), Esmeraldas, Ecuador;; 4 Centre for Tropical Diseases, IRCCS Sacro Cuore Don Calabria Hospital, Verona, Italy

## Abstract

Microscopic examination of stool samples has been considered to be the “gold standard” for diagnosis of intestinal parasites. Recently, polymerase chain reaction (PCR) has been approved by the World Health Organization as the method of choice for the diagnosis of *Entamoeba histolytica*. Of the 106 stool samples collected from the Esmeraldas and Pichincha provinces of Ecuador, all (100%) were positive for *E. histolytica*/*Entamoeba dispar* by light microscopy, whereas using real-time PCR (RT-PCR) DNA amplification, 74 (69.8%) were positive for *E. dispar* and only three (2.8%) were positive for *E. histolytica*. Some 29 (27.4%) samples were negative for the presence of either *E. histolytica* or *E. dispar*, this may be due the presence of *Entamoeba mosksvskii*, which is morphologically identical to *E. histolytica*/*E. dispar* and not specifically targeted by the RT-PCR used. These results indicate the necessity of reevaluating the epidemiology of amebiasis in Ecuador as the prominent species found are nonpathogenic.

Amebiasis is a common intestinal parasitic infection caused by *Entamoeba histolytica*, approximately 500 million people are estimated to be infected worldwide.^1^ The prevalence of *Entamoeba* infection varies being more common in developing countries where poor sanitation leads to ingestion of food or water contaminated with *Entamoeba* spp. cysts. Generally, infections are asymptomatic but in some cases, colitis and extraintestinal infection can occur.^2^

The diagnosis of intestinal amebiasis is based on the microscopic examination of stool samples in search of cyst and/or the trophozoite stages of the parasite. At least six different species of the genus *Entamoeba* can be found in human intestinal lumen; two of them, *Entamoeba dispar* and *Entamoeba moshkovskii*, are morphologically identical to *E. histolytica*, thus making it nigh impossible to differentiate them by microscopy. In addition, *E. histolytica* is the only recognized pathogenic species, although the potential pathogenicity of *E. dispar* and *E. moshkovskii* has not been completely ruled out.^3,4^ Therefore, new diagnostic techniques are needed to differentiate *Entamoeba* species; a rapid diagnostic immunochromatographic antigen test to differentiate *E. histolytica* from the nonpathogenic species exists but has a low specificity for *E. histolytica* sensu stricto.^5^ Molecular DNA-based tools have been shown to be useful to differentiate between *E. histolytica*, *E. dispar*, and *E. moshkovskii*.^2,6^

Previous studies in Ecuador showed a higher prevalence of *E. dispar* infections in comparison with *E. histolytica*.^7,8^ In the present study, 106 samples from patients from the provinces of Esmeraldas (rural area) and Pichincha (urban area) in Ecuador, previously positive for *E. histolytica*/*E. dispar* infection by microscopy, were collected to confirm the presence of infection using real-time polymerase chain reaction (RT-PCR).

A portion of the stool sample was prepared for observation by light microscopy in search of intestinal parasites. Another part of the sample, approximately 200 mg, was preserved in phosphate buffered saline/2% polyvinylpolypyrrolidone and frozen at −20°C till DNA extraction using QIAamp^®^ DNA stool kit (Qiagen N.V., Hilden, Germany) according to the manufacturer’s instructions. In each sample, 2 μL of exogenous Phocine Herpesvirus type-1 DNA (PhHV-1) was added as an internal control. The RT-PCR targets have been described previously by Verweij et al.^9^ Amplification reactions for all the samples were performed in a 25-μL reaction mixture containing PCR buffer (SsoFast master mix; Bio-Rad Laboratories^®^, Milan, Italy), 2.5 μg of bovine serum albumin (Sigma-Aldrich^®^, St. Louis, MO), 80 nM of each of the PhHV-1–specific primers, and 200 nM of PhHV-1 CY5-BHQ2 labeled probe, 60 nM of each *E. histolytica*/*E. dispar* specific primers, and 200 nM of *E. histolytica* fluoroscein amidite-minor grove binder–labeled probe and *E. dispar* 2′-chloro-7′phenyl-1,4-dichloro-6-carboxy-fluorescein-minor grove binder–labeled probe; RT-PCR cycles consisted of 3 minutes at 95°C followed by 40 cycles of 15 seconds at 95°C and 30 seconds at 60°C, and 30 seconds at 72°C. Reactions, detection, and data analyses were performed with the DA7600^®^ RT PCR (DaAn Gene Co. Ltd., Guangzhou, China). Positive and negative controls were included in all the experiments. One of the two positive controls had a low cycle threshold (Ct) (30 < Ct < 36) and the other a high Ct (37 < Ct < 39.9); for all the RT-PCR analysis, the threshold was set at 200.

Using light microscopy, *E. histolytica*/*dispar* cysts were observed in all samples along with other intestinal parasites (Table 1). However, using RT-PCR DNA amplification, few stool samples (*N* = 3 [2.8%]) were positive for *E. histolytica* (Table 2). Given that the method of light microscopy is the standard laboratory method used for diagnosing amoeba, 97.2% of patients are potentially being misdiagnosed and unnecessarily treated. Because of the lack of molecular diagnostics in rural areas, the attending physician must decide if treatment is necessary based more on clinical evidence than laboratory results. The presence of *E. histolytica*/*dispar* cysts in stool samples does not necessarily warrant treatment. In conclusion, a reevaluation of the epidemiology of amebiasis in Ecuador is necessary to minimize these unnecessary treatments for the presence of nonpathogenic species.

**Table 1:** Intestinal parasites in stool samples examined by light microscopy examination

	Eh/d, *n* (%)	Ec, *n* (%)	Ib, *n* (%)	En, *n* (%)	Gl, *n* (%)	Ei, *n* (%)	Enb.i, *n* (%)	Bh, *n* (%)	Tt, *n* (%)	Al, *n* (%)	Sst, *n* (%)
Rural area, *N*											
78	78 (100)	53 (67.9)	5 (6.4)	3 (3.8)	11 (14.1)	6 (7.6)	7 (8.9)	4 (5.1)	26 (33.3)	48 (61.5)	4 (5.1)
Urban area, *N*											
28	28 (100)	11 (39.2)	1 (3.5)	1 (3.5)	–	–	–	3 (10.7)	–	–	–
Total											
106	106 (100)	64 (60.38)	6 (5.6)	4 (3.8)	11 (10.4)	6 (5.6)	7 (6.6)	7 (6.6)	26 (24.5)	48 (45.2)	4 (3.8)

Al = *Ascaris lumbricoides*; Bh = *Blastocystis hominis*; Ec = *Entamoeba coli*; Eh/d = *Entamoeba histolytica/dispar*; Ei = *Entoromonas intestinalis*; En = *Endolimax nana*; Enb.i = *Enbadomonas intestinalis*; Gl = *Giardia lamblia*; Ib = *Iodamoeba butschlii*; Sst = *Strongyloides stercolaris*; Tt = *Trichuris trichiura.* In all cases, cysts were observed except eggs for the helminths Al and Tt. In Sst, the parasite stage detected was larva.

**Table 2:** RT-PCR results

RT-PCR	*Entamoeba histolytica*	*Entamoeba dispar*
	*n* (%)	*n* (%)
Rural area, *n*		
78	0	62 (79.48)
Urban area, *n*		
28	3 (10.71)	12 (42.85)
Total, *n*		
106	3 (2.83)	74 (69.81)

RT-PCR = real-time polymerase chain reaction.

## References

[1] Stanley SL Jr., 2003 Amoebiasis. Lancet 361: 1025–1034. 12660071 10.1016/S0140-6736(03)12830-9

[2] Fotedar R Stark D Beebe N Marriott D Ellis J Harkness J, 2007 Laboratory diagnostic techniques for *Entamoeba* species. Clin Microbiol Rev 20: 511–532. 17630338 10.1128/CMR.00004-07 PMC1932757

[3] Jackson TF, 1998 *Entamoeba histolytica* and *Entamoeba dispar* are distinct species; clinical, epidemiological and serological evidence. Int J Parasitol 28: 181–186. 9504344 10.1016/s0020-7519(97)00177-x

[4] Oliveira FM Neumann E Gomes MA Caliari MV, 2015 *Entamoeba dispar*: could it be pathogenic. Trop Parasitol 5: 9–14. 25709947 10.4103/2229-5070.149887 PMC4327003

[5] Formenti F Perandin F Bonafini S Degani M Bisoffi Z, 2015 Evaluation of the new ImmunoCard STAT!^®^ CGE test for the diagnosis of amebiasis [in French]. Bull Soc Pathol Exot 108: 171–174. 26018388 10.1007/s13149-015-0434-5

[6] Calderaro A Gorrini C Bommezzadri S Piccolo G Dettori G Chezzi C, 2006 *Entamoeba histolytica* and *Entamoeba dispar*: comparison of two PCR assays for diagnosis in a non-endemic setting. Trans R Soc Trop Med Hyg 100: 450–457. 16274714 10.1016/j.trstmh.2005.07.015

[7] Gatti S 2002 Amebic infections due to the *Entamoeba histolytica*-*Entamoeba dispar* complex: a study of the incidence in a remote rural area of Ecuador. Am J Trop Med Hyg 67: 123–127. 12363056 10.4269/ajtmh.2002.67.123

[8] Levecke B Dreesen L Barrionuevo-Samaniego M Benitez Ortiz W Praet N Brandt J Dorny P, 2011 Molecular differentiation of *Entamoeba* spp. in a rural community of Loja province, south Ecuador. Trans R Soc Trop Med Hyg 105: 737–739. 21981992 10.1016/j.trstmh.2011.08.010

[9] Verweij JJ Oostvogel F Brienen EA Nang-Beifubah A Ziem J Polderman AM, 2003 Prevalence of *Entamoeba histolytica* and *Entamoeba dispar* in northern Ghana. Trop Med Int Health 8: 1153–1156. 14641852 10.1046/j.1360-2276.2003.01145.x

